# A randomised controlled trial to prevent hospital readmissions and loss of functional ability in high risk older adults: a study protocol

**DOI:** 10.1186/1472-6963-11-202

**Published:** 2011-08-23

**Authors:** Mary D Courtney, Helen E Edwards, Anne M Chang, Anthony W Parker, Kathleen Finlayson, Kyra Hamilton

**Affiliations:** 1 Faculty of Health and Social Development, University of British Columbia, Okanagan, Canada; 2 School of Nursing and Midwifery, Queensland University of Technology, Brisbane, Australia; 3 Institute of Health and Biomedical Innovation, Queensland University of Technology, Brisbane, Australia; 4 Mater Health Services, Brisbane, Australia; 5 School of Human Movement Studies, Queensland University of Technology, Brisbane, Australia

**Keywords:** Older adults, discharge planning, in-home follow-up, telephone follow-up, exercise, randomised control trial

## Abstract

**Background:**

Older people have higher rates of hospital admission than the general population and higher rates of readmission due to complications and falls. During hospitalisation, older people experience significant functional decline which impairs their future independence and quality of life. Acute hospital services comprise the largest section of health expenditure in Australia and prevention or delay of disease is known to produce more effective use of services. Current models of discharge planning and follow-up care, however, do not address the need to prevent deconditioning or functional decline. This paper describes the protocol of a randomised controlled trial which aims to evaluate innovative transitional care strategies to reduce unplanned readmissions and improve functional status, independence, and psycho-social well-being of community-based older people at risk of readmission.

**Methods/Design:**

The study is a randomised controlled trial. Within 72 hours of hospital admission, a sample of older adults fitting the inclusion/exclusion criteria (aged 65 years and over, admitted with a medical diagnosis, able to walk independently for 3 meters, and at least one risk factor for readmission) are randomised into one of four groups: 1) the usual care control group, 2) the exercise and in-home/telephone follow-up intervention group, 3) the exercise only intervention group, or 4) the in-home/telephone follow-up only intervention group. The usual care control group receive usual discharge planning provided by the health service. In addition to usual care, the exercise and in-home/telephone follow-up intervention group receive an intervention consisting of a tailored exercise program, in-home visit and 24 week telephone follow-up by a gerontic nurse. The exercise only and in-home/telephone follow-up only intervention groups, in addition to usual care receive only the exercise or gerontic nurse components of the intervention respectively. Data collection is undertaken at baseline within 72 hours of hospital admission, 4 weeks following hospital discharge, 12 weeks following hospital discharge, and 24 weeks following hospital discharge. Outcome assessors are blinded to group allocation. Primary outcomes are emergency hospital readmissions and health service use, functional status, psychosocial well-being and cost effectiveness.

**Discussion:**

The acute hospital sector comprises the largest component of health care system expenditure in developed countries, and older adults are the most frequent consumers. There are few trials to demonstrate effective models of transitional care to prevent emergency readmissions, loss of functional ability and independence in this population following an acute hospital admission. This study aims to address that gap and provide information for future health service planning which meets client needs and lowers the use of acute care services.

**Trial Registration No:**

Australian & New Zealand Clinical Trials Registry ACTRN12608000202369

## Background

The ageing of the Australian population presents a significant challenge to the delivery of health services [1]. A significant proportion (13%) of people in Australia is now aged over 65 years [2], with almost one quarter of these older persons suffering from a severe or profound disability [3]. Maintaining functional ability and independence is thus becoming increasingly important to sustain or improve older people's quality of life and contain health care costs. Older people are found to have higher rates of hospital admission and length of stay than the general population. In 2008-09, approximately 3 million separations were recorded by public and private hospitals throughout Australia for older admitted patients (65 years and older), representing 37% of all separations [4]. These rates are comparable to other developed countries where persons aged 65 years and older account for approximately 38% [5] and 36% [6] of hospital admissions in the United Kingdom and United States, respectively.

During hospitalisation older people experience significant functional decline which results in loss of independence, decreased quality of life, and an increased rate of readmission [7-9]. Functional ability is closely tied to quality of life as it is essential for independence in the performance of activities of daily living (ADLs) (e.g., bathing, dressing, transferring, toileting, continence, and feeding) and instrumental activities of daily living (IADLs) (e.g., travelling, shopping, preparing meals, housework, and managing medications, the telephone, and money). One study found that of 1279 community-dwelling patients aged 70 years or over who were admitted to hospital for an acute medical illness, 32% were found to have a decreased ability to perform ADL functions on discharge compared with their pre-admission baseline, with the largest decline occurring in bathing and dressing [9]. At 3 months after discharge, 40% of the study population were found to have a new ADL and/or IADL disability, indicating that functional decline can be long-term. German et al. [10] therefore argue that quality of life can be increased if the rate of hospitalisations for acute episodes can be reduced or eliminated.

The successful move away from institutional-based care has resulted in higher numbers of older adults living at home, often cared for by relatives or spouses of a similar age [11]. Transitional care between hospital and home is required which effectively enables both the older person and their carer, if applicable, to manage at home. Discharge planning aims to improve patient outcomes and contain costs through an in-depth assessment and the development of an individualised plan for the patient prior to leaving hospital. Inadequate assessment of mental and physical status, social and health service support may result in a failure to recognise potential problems which contribute to readmissions. In a systematic review, Shepperd et al. [12] found evidence that discharge planning reduces readmission to hospital and hospital length of stay for older adults with a medical condition. A variety of strategies have been used to strengthen discharge planning, such as early screening, specialised geriatric programs, liaison nurses, and case management [13]. However, strategies are rarely evaluated directly, as outcomes are often assessed by systems measures such as length of hospital stay, rate of readmission and/or costs, rather than focusing on functional ability or psycho-social well-being and of the older person [13].

Evaluations have found readmissions to acute care facilities are decreased with in-home follow-up by nurses [13,14] and that exercise programs, in particular home-based programs, have shown promising results on the health outcomes of older adults [15].

Evidence surrounding case management models for older people, however, remains controversial [16,17]. Lowe and Kasap [18] describe a new model for reorganisation of geriatric and general medical services in a tertiary referral hospital. An iterative process of bed utilisation review, stakeholder consultation, and service remodelling was undertaken to improve bed management and resulted in a reduced length of stay, increased throughput and recurrent cost savings. However, limited work has been undertaken on the promotion of health behaviour change to improve the functional ability of older people at high risk of poor outcomes following discharge in order to improve the maintenance of independence and quality of life.

This research team previously completed a randomised controlled trial investigating the effectiveness of an innovative model of discharge planning and follow-up management of older adults at risk for hospital readmission [19]. The current trial extends this work to determine the effectiveness, as compared to that of receiving usual care, of interventions that 1) combine in-home and telephone follow-up management with hospital-to-home exercise strategies as outlined in the previous trial [19], 2) exercise strategies only intervention, and 3) an in-home and telephone follow-up only intervention. At the writing of this article (December 2010 to March 2011), ethics approval has been obtained and the interventions and data collection from the participants is underway.

### Trial aims

The overall aim of this trial is to evaluate the relative effectiveness of transitional care strategies commencing during hospitalisation for community-based high risk older adults on emergency readmissions and health service use, functional ability and quality of life outcomes. Specifically, the aims are to:

1. Conduct a randomised control trial to compare and evaluate transitional care interventions targeting older patients aged over 65 years who are at risk of hospital readmission after discharge;

2. Compare and evaluate innovative exercise and telephone follow-up interventions following discharge in comparison to usual care, on primary and secondary outcomes at 4 weeks, 12 weeks, and 24 weeks. Primary outcomes include emergency health service use (i.e. unplanned readmissions, time to first unplanned readmission, unplanned Emergency Department, General Practitioner and other health service use), and functional ability. Secondary outcomes include health-related quality of life, psychosocial well-being, and cost effectiveness; and

3. Apply the RE-AIM evaluation framework [20] which uses Reach, Effectiveness, Adoption, Implementation, and Maintenance to assist in understanding the various factors which impact upon the intervention becoming a routine part of the usual care provided by a health service.

## Methods/design

### Study design and setting

The study is a randomised controlled trial to evaluate the effectiveness of exercise-based and/or in-home telephone follow-up strategies for older patients at risk of hospital readmission. Participants are recruited from two tertiary metropolitan hospitals. After baseline data is collected, participants are randomly assigned to one of four groups, either 1) the usual care control group, 2) the exercise and in-home/telephone follow-up intervention group, 3) the exercise only intervention group, or 4) the in-home/telephone follow-up only intervention group.

The usual care control group receives usual discharge planning provided by the health service. The exercise only intervention group, in addition to usual care, receives an assessment by a physiotherapist to form the basis for an individually designed exercise plan aimed at improving functional ability. The program commences in-hospital and is followed up in-home at 6 weekly intervals for 24 weeks by an exercise physiologist. The exercise and in-home/telephone follow-up intervention group, in addition to usual care and the exercise intervention, receives an intervention from admission to 6 months post discharge including home visit/s following hospital discharge and regular telephone follow-up from a gerontology nurse for 6 months post-discharge. The in-home/telephone follow-up only group receives the in-home visits and telephone follow-up from the gerontology nurse as outlines above, with the omission of the exercise program. Participants in all groups complete assessments at baseline (within 72 hours of hospital admission), 4 weeks following hospital discharge, 12 weeks following hospital discharge and 24 weeks following hospital discharge.

### Study sample

#### Recruitment procedures

A hospital-based Research Assistant (RA) recruits participants from two tertiary referral hospitals in Australia. Participants are recruited from four acute medical wards and within 72 hours of admission to hospital. Each day (Monday to Friday), the RA checks the medical wards to determine if there have been any admissions within the last 24 hours (or if Monday morning, over the weekend). If there are any new admissions, the RA will check the patient's medical record to determine if they are eligible to participate in the trial as outlined in the study's inclusion and exclusion criteria (as defined below). If any new patients are eligible to participate, the RA will check that the patient's condition is stable and they are comfortable and, if so, will approach the patient, provide the patient information package, and explain the project. Consent will then be obtained if the patient is interested in participating in the project. If the patient provides consent to participate, the RA collects baseline data. Following collection of baseline data, the RA opens a sealed sequential randomisation envelope, which has been prepared previously by the Project Coordinator via a computerised randomised program, and the participant is randomised to one of the four groups: 1) the usual care control group; 2) the exercise and in-home/telephone follow-up intervention group; 3) the exercise only intervention group; or 4) the in-home/telephone follow-up only intervention group.

#### Inclusion criteria

The inclusion and exclusion criteria reflect patients who are at high risk of hospital readmission after hospital discharge (see [19] for a detailed account of previously published research identifying risk factors for readmission). Participants are included in the trial if the following criteria are met:

• aged 65 years and over,

• admitted with a medical diagnosis (i.e. non-surgical),

• able to speak and understand English, and

• have at least one of the following risk factors for readmissions: - aged 75 years or older,

- hospital admission/s in the previous 6 months to this admission,

- multiple comorbidities (2 or more),

- lives alone,

- lacks social support,

- fair or poor self-rating of health,

- moderate to severe functional impairment, and/or

- history of depression.

#### Exclusion criteria

Participants are excluded from participating in the trial if the following criteria are met:

• they require home oxygen,

• are dependent on a wheelchair or unable to walk independently for 3 meters (patients independently using walking aids are included),

• live in a nursing home (i.e. high dependency unit; patients living in independent hostel or retirement village accommodation are included), or

• have a progressive neurological or cognitive deficit or disease.

#### Randomisation

Participants consenting to participate are randomly allocated into one of the four trial groups after the baseline assessment is completed. A randomisation master list was generated by the project coordinator via a computer-generated randomisation algorithm prior to commencement of recruitment.

#### Sample size

A total of 328 participants (82/group) is aimed to be recruited. Based on our previous research in the area [19] it is anticipated that there will be approximately 25% attrition (20/group) over 6 months of follow-up for reasons such as death, changing mind about participating, or moving away. A sample of 62 completing participants per group (82-20 = 62) is required to detect a 4 week difference in time to hospital readmission. This sample size was determined by power analysis and based on an expected difference in median time to readmission between groups over 24 weeks. Significance level (alpha) was established at 0.05 to avoid a Type 1 error, and power (1 - beta) was set at 90% to avoid a Type II error. Therefore, for a 90% chance of detecting as significant a 4 week difference in time to hospital re-admission, 62 patients in each group are needed to complete the study.

### Blinding

Randomisation to a trial group occurs after baseline assessment is completed, thus the hospital-based RA is initially blind to the study group. A Senior Research Assistant (SRA) is employed (off-site) to enter and collect follow-up outcomes data at 4 weeks following hospital discharge, 12 weeks following hospital discharge, and 24 weeks following hospital discharge. The SRA is blind to the participants' allocated group. An Advanced Practice Gerontic Nurse (APGN) and physiotherapists and exercise physiologist are employed to implement the interventions and it is not be possible to blind the intervention research staff to the trial condition, nor the participants to their allocated intervention.

### Study conditions

#### Usual care control group (Group 1)

Patients who are allocated to the usual care control group receive routine discharge planning and rehabilitation advice as determined by the health service. If the hospital staff identify that in-home visits or rehabilitation services are required after discharge (e.g. community nursing visits), they are organised in the routine manner.

#### Exercise and in-home/telephone follow-up intervention group (Group 2)

Patients who are allocated to the exercise and in-home/telephone follow-up intervention group will receive an intervention following a protocol designed specifically for older patients who are at high risk of poor post-discharge outcomes. The protocol provides a guide for the management and assessment of each patient during the intervention. The intervention extends from recruitment during hospital admission to 24 weeks after discharge and consists of specific patient assessment and management strategies and a schedule of contact visits by the APGN and physiotherapist or exercise physiologist. These include:

Whilst in hospital:

1. APGN, within 72 hours of admission, will visit intervention patients, undertake a health assessment, and prepare a comprehensive transitional care plan. This process includes discussing the planned discharge plan with patient's caregivers, doctor, and ward nurses to individualise the plan;

2. Physiotherapist, within 72 hours of admission, will visit the intervention patients to determine the functional capacity of the patient using measures of ADL and performance tests of balance and gait. This information will be used to plan individualised exercise programs designed to improve strength, stability, coordination, endurance, mobility, and improve self confidence with respect to ADL. The exercise prescription will be developed using a team approach involving the patient, caregivers, doctors, and ward nurses. Goals will be defined for each subject and used as a motivational strategy to improve compliance with the program;

3. Physiotherapist will provide each intervention patient with a pedometer together with simple verbal and written instructions regards usage and journaling of activity levels; and

4. APGN will visit patients 2^nd ^daily thereafter until discharge to establish and implement the program, monitor progress, and modify transitional care plan if required.

After discharge home:

5. APGN, within 48 hours post discharge, provides one in-home visit. This home visit is undertaken to 1) identify sufficient caregiver support is available at home, 2) assess the home environment is safe, 3) determine the patient has the required medications and dressings (if required), 4) ensure the patient (and caregiver) fully understand their medication and treatment regimes; 5) reinforce and further explain exercise program and use of pedometer and journal in the home, and 6) provide advice and support to the caregiver;

6. Additional in-home visits are available by APGN after discharge if required;

7. An exercise physiologist RA conducts 6 weekly in-home visits to reassess the patient's physical measures and functional capacity, evaluate progress with the exercise program, and reset program goals accordingly;

8. APGN, over the initial 4 week period post discharge, provides weekly telephone follow-up calls. Feedback is sought from patients and caregivers regarding medications and treatments and if there are any problems requiring assistance. Where difficulties arise, further advice, information, and support is offered together with reinforcement and further explanation of the exercise program;

9. APGN is available to be contacted via telephone 7 days per week. This availability allows patients and caregivers to access the APGN for further information, support, and guidance should the need arise; and

10. Monthly telephone follow-up calls continue as described above (steps 9 and 10) undertaken by the APGN for 6 months following hospital discharge.

#### Exercise only follow-up intervention group (Group 3)

Patients allocated to the exercise only follow-up intervention group will receive only the exercise component of the protocol, that is, protocol steps 1, 3, 4 and 8.

#### In-home/telephone follow-up only intervention group (Group 4)

Patients allocated to the in-home/telephone follow-up only intervention group

will receive only the APGN component of the protocol, that is, protocol steps 1, 2, 5, 6, 7, 9, 10, and 11.

### Data quality assurance

All completed patient questionnaires are checked for errors and missing data by a SRA as they are returned. Data entry will be double checked against the paper-based questionnaires. Inconsistencies in data entry will be corrected by referring back to the paper-based version of the questionnaire or medical records as appropriate.

### Intervention integrity

The study is guided by the CONSORT (Consolidated Standards of Reporting Trials) statement [21]. Participants will be randomly allocated into one of the four study trial groups after baseline assessment and data collection. Blinding of research staff collecting participant outcomes data will occur. The intervention protocol is documented and all data collected is stored in the study database. Data entry will be double checked against the paper-based versions of the questionnaires. All data analyses will be conducted based on the principle of intention to treat [22].

### Study outcomes

#### Primary outcomes

Primary outcomes include emergency health service use (i.e. unplanned readmissions, time to first unplanned readmission, unplanned Emergency Department, General Practitioner and other health service use), and functional ability (Instrumental Activities of Daily Living [23], Index of Activities of Daily Living [24], Walking Impairment Questionnaire [25]).

#### Secondary outcomes

Secondary outcomes include health-related quality of life (Short Form-12^v2 ^Survey [26]), psychosocial well-being (Geriatric Depression Scale [27], MOS Social Support Survey [28]), satisfaction with health care, and cost effectiveness (i.e., calculating the number of readmissions avoided, estimating time gained by delaying or preventing time to first re-admission, calculating mean treatment cost differentials between control and intervention groups).

#### Intervention Implementation

Adherence to the program is assessed and recorded during each follow-up implementation visits and/or telephone follow-up calls, including patients' exercise adherence and motivation.

#### Socio-demographic Information

Self-reported socio-demographic factors include age (in years), sex (male or female), ethnicity (born in Australia or overseas), education (attendance at secondary school or greater, employment status (currently employed or not), income levels (annual income <$30 K, $30-60 K or >$60 K), living arrangements (live with relative/friend, or spouse or live alone), and hospital insurance status (Medicare, private insurance, or Department of Veteran Affairs).

### Data analysis

#### Descriptive analysis

Baseline data from the control and intervention groups will be compared to check for comparability of the groups. Chi square tests will be used for categorical variables, ANOVA for normally distributed continuous variables, and Kruskal-Wallis tests for non-parametric data. Differences detected will be controlled for during the subsequent analyses.

#### Primary and secondary analysis

All data analyses will be conducted based on the principle of intention to treat [22]. Chi square, ANOVA, and Kruskal-Wallis tests will be used for bivariate analysis. For the primary outcomes of emergency health service use, Kaplan-Meier survival curves and log rank statistic will be used to compare intervention and control groups. A Cox proportional hazards regression model will be used to determine the effect of the interventions and adjust for any potential confounding variables. A general linear model approach will be used for multivariable repeated measures analysis of covariance for continuous outcome variables (e.g. measures of functional status, quality of life and psycho-social well-being) to investigate the differences over the four data collection points. Economic data will be collected to enable a comprehensive evaluation of the cost-effectiveness of the interventions when compared to that of usual care. Mean treatment costs differentials between control and intervention groups will be calculated. The patient level data will be used alongside other routinely available data to inform a decision analytic cost-effectiveness model.

### Publication process

The principal and co-investigators of the grant will be responsible for ensuring the results of the trial, regardless of the outcomes, are published within a reasonable timeframe after conclusion of the trial. The reporting of this trial will adhere to the latest CONSORT [21] statements at the time of manuscript submission.

### Ethical approval

The trial has received ethical approval from the Human Research Ethics Committees of the Mater Health Services (No. 1173A) and Queensland University of Technology (No. 0800000219). The trial will be implemented in compliance with this protocol and the Australian National Statement on Ethical Conduct in Human Research [29].

## Discussion

Acute hospital services comprise the largest section of health expenditure in developed countries. Prevention or delaying of disease is known to produce more effective use of services and determining the optimal transitional care for at-risk patients following a hospital admission has still not been achieved. Lowering the use of acute care services is the most beneficial and significant cost saving expected as a result of positive outcomes from the proposed interventions in this study. This knowledge will permit forward planning and the development and construction of effective health services to meet client needs and prevent emergency readmissions into hospital.

A limited number of long term trials have been undertaken on interventions to prevent emergency health service use and promote health behaviour changes to improve the functional ability, independence and quality of life of older people at high risk of poor outcomes following hospital discharge. Accordingly, current models of discharge planning and follow-up care do not always address their needs. In a recent randomised control trial [19], support was found for the effectiveness of an innovative model of discharge planning and follow-up management of older adults at risk for hospital readmission; however, the trial was unable to examine the optimal components of the multi-faceted intervention on health and economic outcomes. This current trial extends this previous work to determine the comparative effectiveness, as compared to that of receiving usual care, of interventions that deliver 1) in-home and telephone follow-up management with hospital-to-home exercise strategies, 2) exercise strategies only, and 3) in-home and telephone follow-up only. The results will determine effective strategies to reduce readmissions and improve functional status, independence, and psycho-social well-being in this at risk group.

## Competing interests

The authors declare that they have no competing interests.

## Authors' contributions

MDC, HEE, AMC, AWP, KF conceptualised the trial and design and secured funding. All authors contributed to development and revision of the manuscript and take public responsibility for its content. All authors have read and approved the final manuscript.

## Pre-publication history

The pre-publication history for this paper can be accessed here:

http://www.biomedcentral.com/1472-6963/11/202/prepub
